# Editorial: Principles Underlying Post-Stroke Recovery of Upper Extremity Sensorimotor Function – A Neuroimaging Perspective

**DOI:** 10.3389/fneur.2015.00267

**Published:** 2015-12-23

**Authors:** Bruno J. Weder, Roland Wiest, Rüdiger J. Seitz

**Affiliations:** ^1^Support Center for Advanced Neuroimaging (SCAN), Institute for Diagnostic and Interventional Neuroradiology, University Hospital Inselspital, University of Bern, Bern, Switzerland; ^2^Department of Neurology, Kantonsspital St. Gallen, St. Gallen, Switzerland; ^3^Department of Neurology, Centre of Neurology and Neuropsychiatry, LVR-Klinikum Düsseldorf, Heinrich-Heine-University Düsseldorf, Düsseldorf, Germany; ^4^Florey Institute of Neuroscience and Mental Health, University of Melbourne, Parkville, VIC, Australia

**Keywords:** stroke recovery, multimodal neuroimaging, computational biophysical modeling, motor control, motor imagery, somatosensory disorders, perilesional plasticity, network reorganization

**The Editorial on the Research Topic**

**Principles Underlying Post-Stroke Recovery of Upper Extremity Sensorimotor Function – A Neuroimaging Perspective**

A substantial proportion of stroke survivors suffer from long-term sensorimotor deficits of the contralesional arm and hand (1). Neuroimaging, using a diversity of methods, has the potential to uncover underlying principles of functional disabilities and recovery characterizing patient groups as well as individual variability (2–6). The present issue aims at (i) revealing the physiological mechanisms and the long-term course of stroke recovery with respect to site and size of lesions, (ii) correlating behavioral deficits and electrophysiological parameters with imaging patterns, (iii) delineating neural networks involved, and (iv) identifying sites where interventions enhance the recovery process.

Seitz and Donnan give an overview of mechanisms and disease-related limitations in post-stroke recovery. They address two informative subsections delineating time courses of the recovery process and state-of-the-art of neurorehabilitative training to improve the stroke-induced neurological deficit.

Auriat et al. complete this clinical perspective with an overview on the use of transcranial magnetic stimulation and multimodal neuroimaging to estimate functional resources post-stroke. They provide a review of data from studies utilizing DTI, MRS, fMRI, EEG, and brain stimulation techniques, focusing on TMS and its combination with uni- and multimodal neuroimaging methods with respect to their benefits and limitations.

Falcon et al. used “The Virtual Brain (TVB),” an open source platform based on local biophysical models. Using this platform, they simulated individuals’ brain activity linking structural data directly to a TVB model. Correlating TVB parameters with graph analysis metrics, they obtained evidence for a shift of global to local dynamics in chronic stroke patients.

Buetefisch reviews the role of an intact contralesional motor cortex (M1) in post-stroke recovery of upper extremity motor function. The impact of the contralesional M1, on the lesioned motor cortex, seems to be promoting activity in the acute and inhibiting it in the chronic stage. Supportive evidence comes from animal studies, including changes in neurotransmitter systems, dendritic growth, and synapse formation. Thus, the contralesional M1 may represent a treatment target during rehabilitation.

Sharma and Baron report an fMRI study of a finger-thumb opposition sequence in chronic, well-recovered subcortical stroke patients. Using independent component analysis, they could show that recovery of motor function involved pre-existing cortical networks contributing to recovery in a differentiated manner.

The study of Abela et al. complements these investigations of functional networks associated with recovery in the case of cortical sensorimotor stroke. The structural covariance network in patients recovering from hand paresis encompassed (i) a cortico-striato-thalamic loop involved in motor execution and (ii) higher order sensorimotor cortices affected by the stroke lesions. The network emerged in the early chronic stage post-stroke was related to gray matter volume increases in the ipsilesional medio-dorsal thalamus, and its expression depend on an interaction of recovered hand function and the lesion size.

Bannister et al. report about neuroimaging evidence for the significance of the contralesional hemisphere in the recovery process after hemispheric supratentorial ischemic stroke, thus supplementing the review of Buetefisch. They followed the time course of touch sensation in the upper extremity using resting state – fMRI to explore functional connectivity. Improvement of touch sensation was related to changes in the contralesional hemisphere and cerebellum: (1) an increase in connectivity strength between the secondary somatosensory area seed and both inferior parietal cortex and middle temporal gyrus as well as the thalamus seed and cerebellum and (2) a decrease in connectivity strength between SI seed and the cerebellum.

Primaßin et al. dealed with four exemplary cases in which motor and language domains were affected differently. They focused on dissociative outcomes after 7 weeks of rehabilitative treatment following the predominant failure at baseline. Primarily, precise location of the lesions in the corticospinal tract and/or fasciculus arcuatus, respectively, turned out to be critical for recovery. Motor and language improvement seemed to occur together, rather than to compete for recovery resources.

Ben-Shabat et al. investigated changes in human proprioception, its specific brain activation, laterality, and changes following stroke. Brain activation involved the supramarginal gyrus (SMG) and dorsal premotor cortex (PMd) with a prominent lateralization in the former. Lateralization was diminished in three patients exhibiting proprioceptive deficits post-stroke and a common lesion within the thalamus. The findings underline the role of SMG and dPM in spatial processing and motor control.

Brugger et al. investigated the intriguing role of supplementary motor complex (SMC) and disturbed motor control, a retrospective clinical and lesion analysis of 10 patients presenting anterior cerebral artery stroke. In the very acute phase, alien hand syndrome (AHS) dominated accompanied by failed conscious awareness of motor intention and a missing sense of agency while performing externally triggered movements. In the follow-up, motor signs specifically related to AHS, i.e., disturbed self-initiated movements, grasping, and intermanual conflict, were mainly related to lesions of the pre-supplementary motor area and medial cingulate cortex.

Camilleri et al. studied the neural substrate underlying the performance of the trail making test (TMT) that is often used in the follow-up of stroke. In healthy volunteers, they found that performance in terms of motor speed to be related to the local brain volume of a region in the lower bank of the left inferior sulcus. Conjunction analysis of four connectivity approaches has shown this area to represent a constituent of the so-called multiple demand network, highlighting the TMT as related rather to executive than primary motor function.

In summary, the neurological deficits, recovery mechanisms, and the prognosis for recovery after stroke are hot spots of clinical neurology and systems neuroscience research. Multimodal imaging, applied neurophysiology, and careful neurobehavioral *in vivo* correlations have opened new vistas on the pathophysiological mechanisms underlying post-stroke recovery of upper extremity sensorimotor deficits paving new avenues for future research.

## Conflict of Interest Statement

The authors declare that the research was conducted in the absence of any commercial or financial relationships that could be construed as a potential conflict of interest.
